# Targeting SphK2 Reverses Acquired Resistance of Regorafenib in Hepatocellular Carcinoma

**DOI:** 10.3389/fonc.2020.00694

**Published:** 2020-06-24

**Authors:** Weiwei Shi, Shan Zhang, Ding Ma, Dongliang Yan, Guang Zhang, Yin Cao, Zhongxia Wang, Junhua Wu, Chunping Jiang

**Affiliations:** ^1^Department of Hepatobiliary Surgery, The Affiliated Drum Tower Hospital of Nanjing University Medical School, Nanjing, China; ^2^Jiangsu Key Laboratory of Molecular Medicine, Medical School, Nanjing University, Nanjing, China; ^3^Department of Hepatobiliary Surgery, Drum Tower Clinical College of Nanjing Medical University, Nanjing, China

**Keywords:** sphingosine kinase 2, sphingosine-1-phosphate, ABC294640, regorafenib, resistance, hepatocellular carcinoma

## Abstract

**Background:** Regorafenib is a second-line therapy drug used for advanced hepatocellular carcinoma (HCC). Unfortunately, the survival benefit of the patients receiving this treatment is modest, which may be attributed to drug resistance. In the present study, sphingosine kinase 2 (SphK2) was targeted to reverse regorafenib resistance in HCC.

**Methods:** The functions of SphK2 and sphingosine-1-phosphate (S1P), the catalytic product of SphK2 in regorafenib resistance of HCC cells, were evaluated by cell counting kit-8 assay, colony formation, cell cycle evaluation, and annexin V–fluorescein isothiocyanate/propidium iodide double-staining assay. The antitumor activity of combined treatment of regorafenib and the SphK2-specific inhibitor ABC294640 was examined in HCC cells *in vitro* and xenograft model *in vivo*. The molecular mechanisms of SphK2/S1P-mediating regorafenib resistance were investigated using cell line establishment and Western blot analysis.

**Results:** Well-developed regorafenib-resistant HCC cells indicated high expression levels of SphK2. The sensitivity to regorafenib of regorafenib-resistant HCC cells was restored following SphK2 knockdown or pharmacological inhibition by ABC294640. In addition, ectopic expression of SphK2 and exogenous addition of S1P decreased the sensitivity of HCC cells to regorafenib. Furthermore, the combination treatment with ABC294640 sensitized resistant tumor to regorafenib in xenograft model of HCC. The phosphorylation levels of nuclear factor κB (NF-κB), as well as those of signal transducer and activator of transcription 3 (STAT3), were positively associated with SphK2 and S1P.

**Conclusions:** SphK2/S1P mediates regorafenib resistance of HCC through NF-κB and STAT3 activation. Targeting SphK2 by ABC294640 potently reduces regorafenib resistance of HCC cells both *in vitro* and *in vivo*. The combination of ABC294640 and regorafenib could be developed as a novel potential treatment strategy for advanced HCC.

## Introduction

With 841,000 newly diagnosed cases and 782,000 deaths annually, liver cancer ranked as the sixth most common cancer and the fourth leading cause for cancer-related deaths worldwide (1). Hepatocellular carcinoma (HCC), which accounts for 75 to 85% cases of primary liver cancer, is the most common pathological type of this deadly disease (2).

The majority of HCC patients are diagnosed at an advanced stage, which limited the applicability and efficacy of potentially curative therapies, including surgical resection and liver transplantation (3). Therefore, patients with advanced HCC rely mainly on systemic therapy. Approved by the US Food and Drug Administration in April 2017, regorafenib is currently used as a second-line systemic therapy for advanced HCC (4). Similar with the first-line systemic drug sorafenib, regorafenib is also an oral tyrosine kinase inhibitor that targets vascular endothelial growth factor receptors (VEGFR1, VEGFR2, and VEGFR3), platelet-derived growth factor receptor, Fibroblast Growth Factor Receptor 1, Raf, TIE-2, and the kinases KIT, RET, and BRAF (5). Despite the fact that regorafenib prolonged the survival of patients who had disease progression following sorafenib failure, the efficacy of this drug was still limited by primary or acquired therapy resistance (6). Therefore, it is essential to investigate the mechanisms underlying the resistance to regorafenib and to further explore strategies to enhance drug efficacy in HCC (7).

Sphingosine kinases (SphKs) are the key regulatory enzymes catalyzing the formation of sphingosine-1-phosphate (S1P) (8). With increasing evidence of the roles of SphKs in cell survival, proliferation, apoptosis, and chemoresistance, these enzymes are considered as significant therapeutic targets in various solid tumors (9). To date, SphK1 and SphK2 have been identified as the two isoforms of SphKs. Considerable attention has been devoted to the involvement of SphK1 in multiple cancers including HCC (10). Recently, the other isoform of SphK, SphK2, also received increasing attention and may be an important regulator of cancer development and progression (11). Accumulating evidence has revealed that SphK2 is overexpressed in tumor tissues and cell lines (12, 13). Knockdown or pharmacological inhibition of SphK2 can decrease tumor proliferation and metastasis and increase apoptosis *in vivo* and *in vitro* (14, 15). It is interesting to note that data from certain studies have shown that SphK2 is closely associated with antitumor drug resistance. Overexpression of SphK2 has been suggested to contribute to gefitinib resistance in non-small cell lung cancer (NSCLC) and all-*trans* retinoic acid (ATRA) resistance in colon cancer (13, 16). However, whether SphK2 is involved in regorafenib resistance in HCC remains unclear.

ABC294640 is a highly selective and orally available small molecule inhibitor of SphK2 that can dose-dependently compete with sphingosine for binding to the enzyme. ABC294640 displayed significant antitumor activity in various solid cancers, including breast (17), lung (15), prostate (18), and liver (19) cancers. Currently, ABC294640 is under evaluation in a phase II clinical trial as a therapy for advanced HCC. Administration of ABC294640 can further enhance the effects of antitumor drugs including sorafenib (20). By coadministration of ABC294640, the potency of sorafenib in HCC, cholangiocarcinoma, pancreatic adenocarcinoma, and kidney carcinoma cells was increased (21). Therefore, it is interesting to investigate whether ABC294640 could also enhance the effects of regorafenib and even reverse regorafenib resistance in HCC.

In the present study, we explored the role and potential molecular mechanisms of SphK2 in regorafenib-resistant HCC cells. ABC294640 was used to investigate the efficacy of targeting SphK2 for reversing regorafenib resistance *in vitro and vivo*. The study aimed to provide experimental evidence for the clinical application of ABC294640 in combination with regorafenib.

## Results

### Acquired Resistance Develops After Long-Term Exposure to Regorafenib

**Figure 1:**
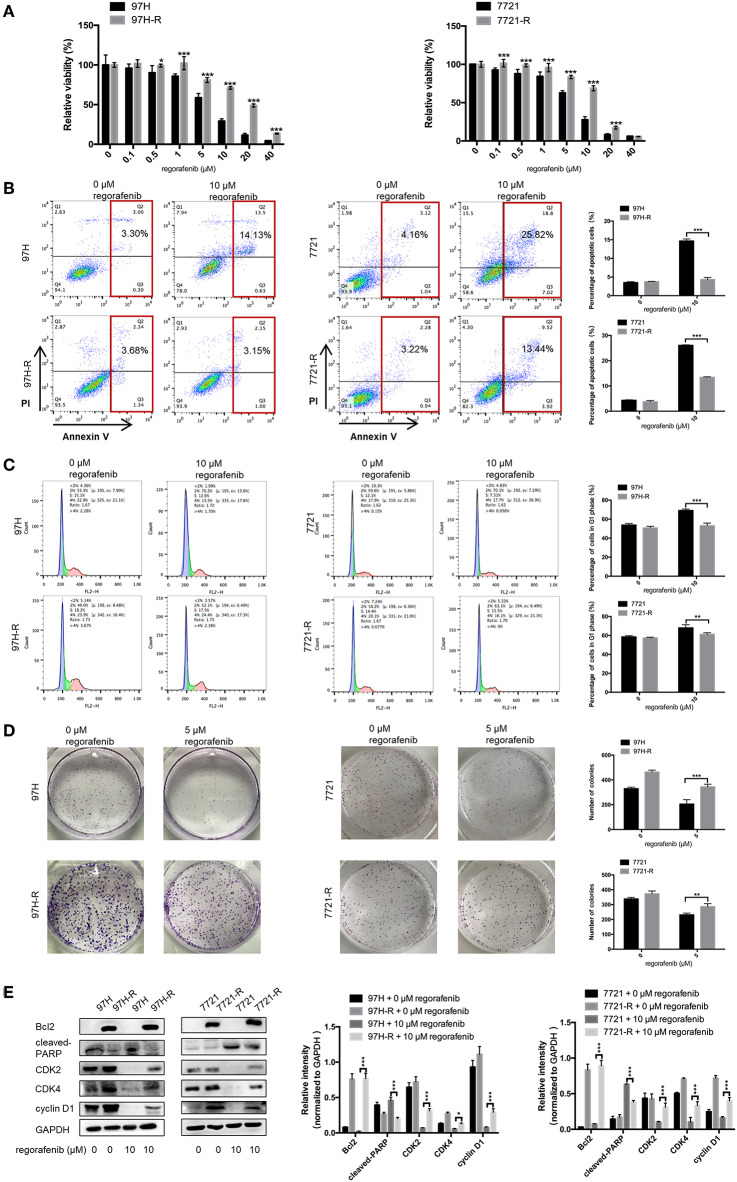
Establishment of regorafenib-resistant HCC cells. **(A)** The CCK-8 assay was used to compare the effects of regorafenib on cell proliferation between parental and regorafenib-resistant HCC cells. **(B)** The percentage of apoptotic parental and regorafenib-resistant HCC cells treated with or without 10 μM regorafenib for 48 h was determined by annexin V/PI staining. **(C)** The cell cycle distribution of parental and regorafenib-resistant HCC cells treated with or without 10 μM regorafenib for 48 h was detected by flow cytometry. **(D)** The colony formation activity and the cell proliferation of parental and regorafenib-resistant HCC cells treated with or without 5 μM regorafenib (14 days for SMCC-7721 and 7721-R; 10 days for MHCC-97H and 97H-R, respectively) were measured. **(E)** The expression levels of Bcl2, cleaved PARP, cyclin D1, CDK2, and CDK4 were examined by Western blot analysis. 7721 and 97H indicate SMMC-7721 and MHCC97H parental cells, respectively; 7721-R and 97H-R indicate regorafenib-resistant SMMC-7721 and regorafenib-resistant MHCC97H cells, respectively. The result is representative for three independent experiments. The error bars represent mean ± SD from a representative experiment. *p < 0.05, **p < 0.01, ***p < 0.001.

The SMMC-7721 and MHCC97H cell lines were used to establish cell lines resistant to regorafenib. After the establishment of the resistant cell lines, we characterized their resistant phenotype. Initially, the CCK-8 assay was used, and the data demonstrated that the growth-suppressive effect of regorafenib was significantly higher in parental cells than in regorafenib-resistant cells (Figure 1A). The IC_50_ values for regorafenib (Table 1) were considerably higher in resistant cells (97H-R: 16.85 μM, 7721-R: 12.27 μM) than in parental cells (97H: 5.378 μM, 7721: 5.431 μM). In addition, the total percentage of apoptotic regorafenib-resistant cells treated with 10 μM regorafenib was significantly lower than that of the parental cells as shown by flow cytometry analysis (Figure 1B, *p* < 0.001). Cell cycle analysis demonstrated that regorafenib induced G1 phase arrest in parental cells but not in regorafenib-resistant cells at a dose of 10 μM (Figure 1C). We also observed using a colony formation assay that the proliferative potential of regorafenib-resistant cells treated with or without 5 μM regorafenib was significantly higher than that of parental cells (Figure 1D). In addition, the differential effects of regorafenib in parental and regorafenib-resistant cells were confirmed by measurement of the expression levels of two apoptotic cascade-related proteins, B-cell leukemia/lymphoma 2 (Bcl2) and poly(ADP-ribose) polymerase (PARP). The effect of regorafenib on cell proliferation was also verified by the expression of cyclin D1 and cyclin-dependent kinase 2 and 4 (CDK2, CDK4). These results indicated that the regorafenib-resistant cells showed less response to regorafenib exposure as compared to parental cells (Figure 1E). Collectively, our data confirmed the establishment of stable regorafenib-resistant cells.

**Table 1:** IC_50_ values of regorafenib in parental and regorafenib-resistant HCC cells.

**Cell line**	**IC_**50**_ (μM)**
97H	5.378
97H-R	16.85
7721	5.431
7721-R	12.27

### SphK2 Expression Is Negatively Associated With Regorafenib Sensitivity in HCC Cells and Is Upregulated in Regorafenib-Resistant HCC Cells

**Figure 2:**
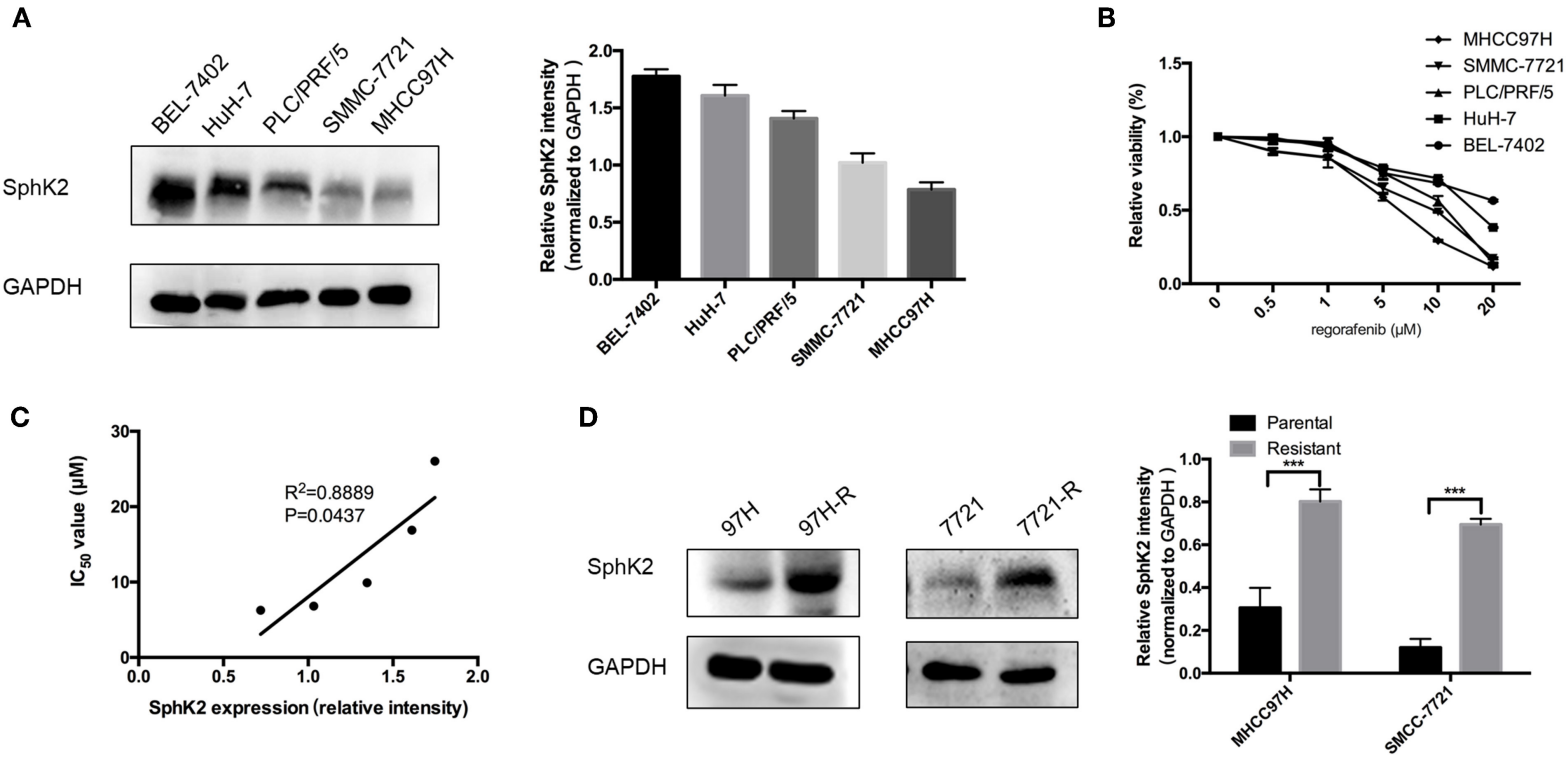
Association between SphK2 expression and regorafenib sensitivity in HCC cells. **(A)** The protein expression of SphK2 in five HCC cell lines was determined by Western blot analysis. **(B)** Hepatocellular carcinoma cells were incubated with regorafenib for 48 h, and regorafenib sensitivity was determined by the CCK-8 assay. **(C)** The correlation between SphK2 expression and the regorafenib IC_50_ value in HCC cell lines was assessed by the Pearson correlation coefficient. **(D)** SphK2 protein expression in parental and regorafenib-resistant HCC cells. The result is representative for three independent experiments. The error bars represent mean ± SD from a representative experiment. ***p < 0.001.

To investigate the potential involvement of SphK2 in regorafenib resistance, five HCC cell lines were used, namely, BEL-7402, HuH-7, PLC/PRF/5, SMMC-7721, and MHCC97H. The characteristics of these cells including the origin, morphology, doubling time, tumorigenicity, metastatic potential, and cellular products are summarized in Table 2 (22–26). Among these cell lines, PLC/PRF/5 is the only one originated from African male, and BEL-7402 grows the fastest. MHCC97H was reported to develop massive lung metastasis when inoculated subcutaneously and is considered to have high metastasis potential, whereas HuH-7 is considered to be a non-invasive cell line. Hepatitis B surface antigen, which is known to be associated with the development of HCC, is expressed in PLC/PRF/5 and MHCC97H. The α-fetoprotein (α-FP), which represents the aggressiveness of HCC cells, is expressed in all cell lines except SMMC-7721. Based on the information, no obvious correlation was found between the characteristics and SphK2 expression levels. We evaluated SphK2 expression, as well as regorafenib sensitivity in these cells. As shown in Figures 2A,B, the protein levels of SphK2 were apparently higher in HCC cells with higher regorafenib IC_50_ values (Table 3). In addition, the SphK2 protein levels and the IC_50_ values exhibited a strong correlation, with a Pearson correlation coefficient (*R*^2^) of 0.8889 (Figure 2C), indicating that SphK2 expression was negatively associated with regorafenib sensitivity in HCC cell lines. Furthermore, SphK2 was significantly upregulated in regorafenib-resistant cells compared with the corresponding expression noted in parental cells (Figure 2D), whereas the expression of SphK1 remained unchanged (Supplementary Figure 1A), suggesting that SphK2 expression was positively associated with regorafenib resistance.

**Table 2:** General characteristics of 5 HCC cell lines.

**Cell line**	**Origin**	**Cell morphology**	**Doubling time**	**Tumorigenicity**	**Metastatic potential**	**HBsAg**	**α-FP**
BEL-7402	Human HCC Asian male	Epithelial	20 h	Yes	Low	Neg	Pos
HuH-7	Human HCC Asian male	Epithelial	38 h	Yes	Non	Neg	Pos
PLC/PRF/5	Human HCC African male	Epithelial	43 h	Yes	Low	Pos	Pos
SMMC-7721	Human HCC Asian male	Epithelial	40 h	Yes	Low	Neg	Neg
MHCC97H	Human HCC Asian male	Epithelial	31 h	Yes	High	Pos	Pos

*HBsAg, hepatitis B surface antigen; α-FP, α-fetoprotein; Neg, negative; Pos, positive*.

**Table 3:** IC_50_ values of regorafenib in 5 HCC cell lines.

**Cell line**	**IC_**50**_ (μM)**
BEL-7402	26.04
HuH-7	16.90
PLC/PRF/5	9.91
SMMC-7721	6.81
MHCC97H	6.26

### Overexpression of SphK2 Promotes Regorafenib Resistance in HCC Cells

**Figure 3:**
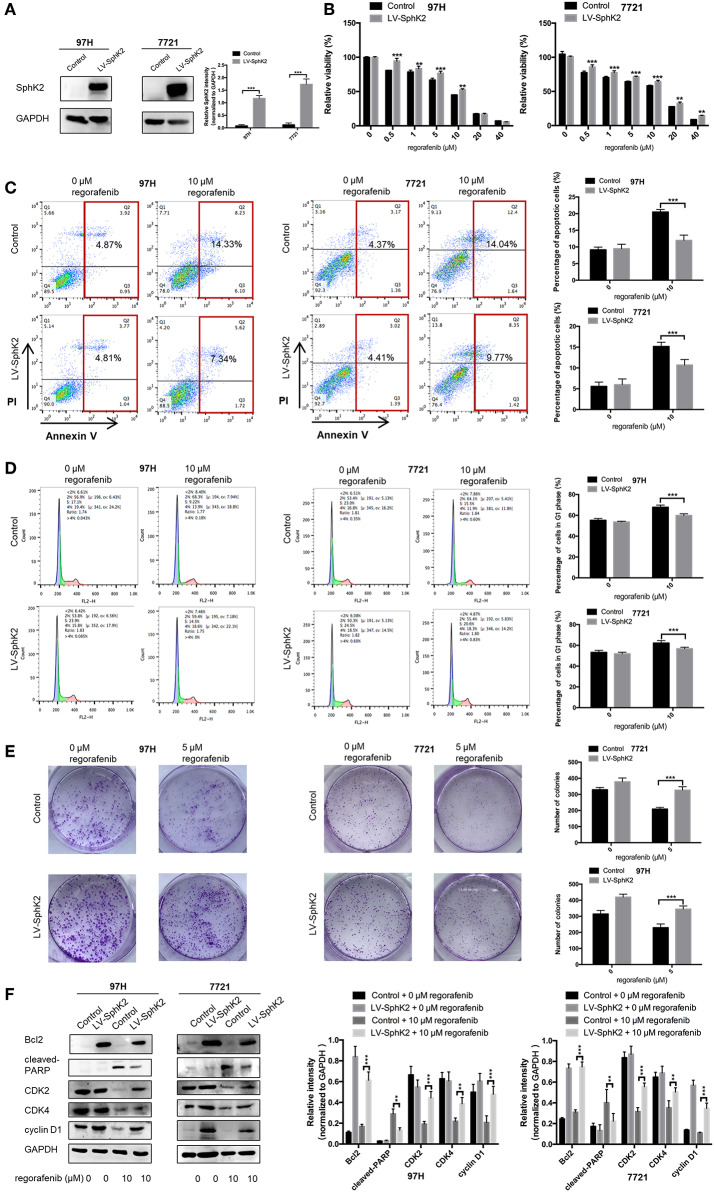
Effects of SphK2 overexpression on the sensitivity of HCC cells to regorafenib. **(A)** The protein expression of SphK2 in HCC cells transfected with LV-SphK2 lentivirus or control vector was measured by Western blot analysis. **(B)** Regorafenib sensitivity of HCC cells transfected with LV-SphK2 lentivirus or control lentivirus was assessed by the CCK-8 assay, and regorafenib IC_50_ values were calculated accordingly. **(C)** The annexin V–FITC/propidium iodide double-staining assay, **(D)** cell cycle analysis, and **(E)** colony formation assay were applied to compare regorafenib sensitivity of HCC cells in the two different groups. **(F)** The expression levels of Bcl2, cleaved PARP, cyclin D1, CDK2, and CDK4 were examined by Western blot analysis. The dose of regorafenib treatment in the assays was 10 μM for 48 h, with the exception of the colony formation assay (5 μM, 14 days for SMCC-7721 and 10 days for MHCC-97H, respectively). The result is representative for three independent experiments. The error bars represent mean ± SD from a representative experiment. **p < 0.01, ***p < 0.001.

To further investigate the role of SphK2 in promoting regorafenib resistance in HCC, SMMC-7721 and MHCC97H HCC cells were stably transfected with LV-SphK2 lentivirus to enhance the expression of SphK2. The increased expression of SphK2 in these two cell lines compared with that in the control cells was confirmed by Western blot analysis (Figure 3A). SphK2-overexpressing HCC cells (LV-SphK2 HCC cells) exhibited low sensitivity to regorafenib, as determined by the CCK-8 assay (Figure 3B), which was similar to the results demonstrated in HCC cells with acquired regorafenib resistance. The IC_50_ values of SphK2-overexpressing HCC cells were higher than those of the cells in the control group (Table 4). In LV-SphK2 HCC cells, exposure to 10 μM regorafenib for 48 h exhibited a limited impact on the percentage of apoptotic cells (Figure 3C) and number of G1-phase arrested cells (Figure 3D). The proliferative potential of LV-SphK2 cells treated with or without 5 μM regorafenib was significantly higher than that of control cells, as determined by the colony formation assay (Figure 3E). In addition, overexpression of SphK2 reversed the changes in Bcl2, cleaved PARP, cyclin D1, CDK2, and CDK4 (Figure 3F) expression following incubation of the cells with regorafenib.

**Table 4:** IC_50_ values of regorafenib in SphK2-overexpressing HCC cells and control group cells.

**Cell line**	**Group**	**IC_**50**_ (μM)**
97H	Control	6.145
	LV-SphK2	9.592
7721	Control	6.36
	LV-SphK2	10.22

### Knockdown of SphK2 Restores Regorafenib Sensitivity in Regorafenib-Resistant HCC Cells

**Figure 4:**
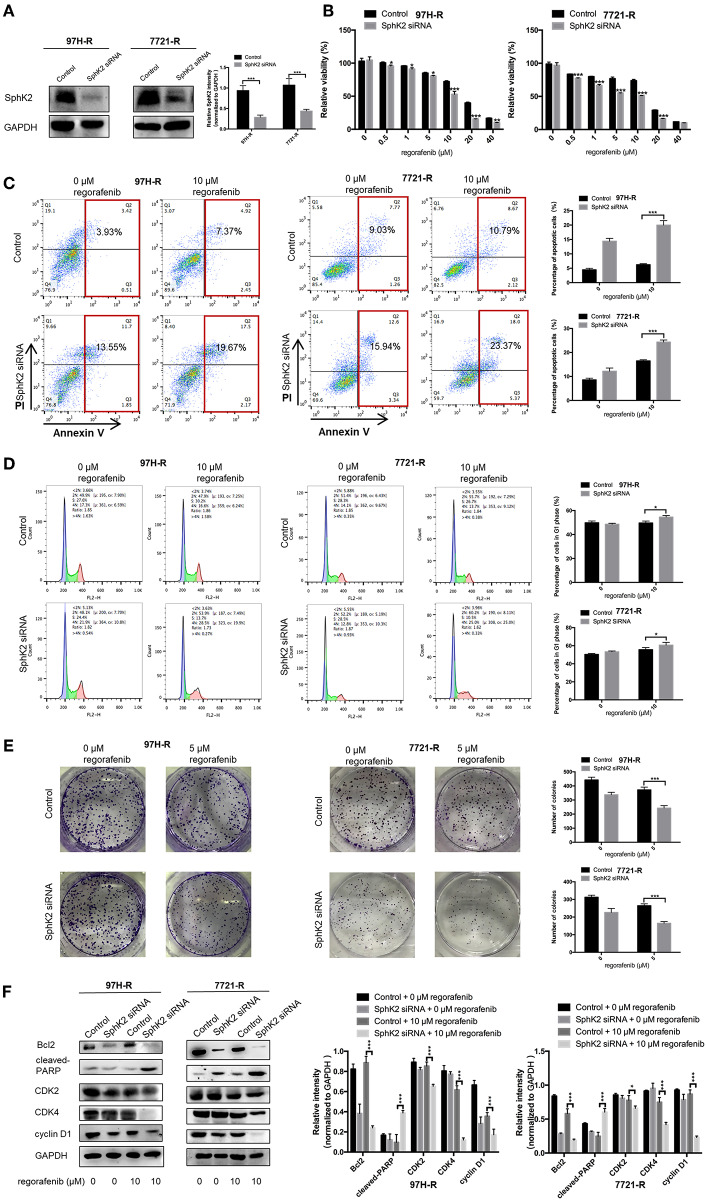
Effects of SphK2 knockdown on regorafenib resistance in HCC cells. **(A)** The protein expression levels of SphK2 in HCC cells transfected with SphK2 siRNA or control vector were determined by Western blot analysis. **(B)** The viability of regorafenib-resistant HCC cells transfected with SphK2 siRNA or control vector was determined by the CCK-8 assay, and IC_50_ values were calculated accordingly. **(C)** The induction of apoptosis of regorafenib-resistant HCC cells transfected with SphK2 siRNA or control vector was assessed by the annexin V–FITC/propidium iodide double-staining assay. **(D)** The cell cycle distribution of regorafenib-resistant HCC cells transfected with SphK2 siRNA or control vector was assessed by flow cytometry. **(E)** The proliferation of regorafenib-resistant HCC cells transfected with SphK2 siRNA or control vector was evaluated by the colony formation assay. **(F)** The expression levels of Bcl2, cleaved PARP, cyclin D1, CDK2, and CDK4 were examined by Western blot analysis. The dose of regorafenib treatments in all assays was 10 μM for 48 h, except for the colony formation assay (5 μM, 14 days for 7721-R and 10 days for 97H-R, respectively). The result is representative for three independent experiments. The error bars represent mean ± SD from a representative experiment. *p < 0.05, **p < 0.01, ***p < 0.001.

Because the previous results suggested that enhanced SphK2 expression may be a cause of acquired resistance to regorafenib in HCC cells, we attempted to knock down SphK2 in 97H-R and 7721-R cells to determine whether regorafenib resistance could be reversed. SphK2 small interfering RNA (siRNA) transfection resulted in significantly decreased expression of SphK2 in both regorafenib-resistant cell lines, as verified by Western blot analysis (Figure 4A). Transfection of SphK2 siRNA into HCC cells enhanced the inhibitory effect of regorafenib on the viability of regorafenib-resistant cells (Figure 4B), as the IC_50_ values of SphK2-knockdown regorafenib-resistant cells were lower than those of control group cells (Table 5). In contrast, siRNA-mediated knockdown of SphK1 (Supplementary Figure 1B) showed little impact on the sensitivity of regorafenib (Supplementary Figure 1C) and the IC_50_ values (Supplementary Table 1) in both resistant cell lines. The apoptosis assay performed on regorafenib-resistant cells demonstrated that incubation with regorafenib (10 μM) alone induced apoptosis only slightly, and SphK2 knockdown mildly increased the percentage of apoptotic cells. However, the combined effect of the two treatments was superior to the effect of either treatment alone (Figure 4C). Similar results were found in the cell cycle (Figure 4D) and colony formation assays (Figure 4E). Furthermore, the combination of SphK2 knockdown and regorafenib decreased Bcl2, cyclin D1, CDK2, and CDK4 expression levels and increased cleaved PARP expression levels. These results indicated that SphK2 knockdown successfully enhanced the effects of regorafenib and restored regorafenib sensitivity in regorafenib-resistant HCC cells.

**Table 5:** IC_50_ values of regorafenib in SphK2 knockdown HCC cells and control group cells.

**Cell line**	**Group**	**IC_**50**_ (μM)**
97H-R	Control	15.24
	SphK2 siRNA	10.08
7721-R	Control	11.13
	SphK2 siRNA	6.243

### Exogenous Addition of S1P Increases the Resistance of HCC Cells to Regorafenib

**Figure 5:**
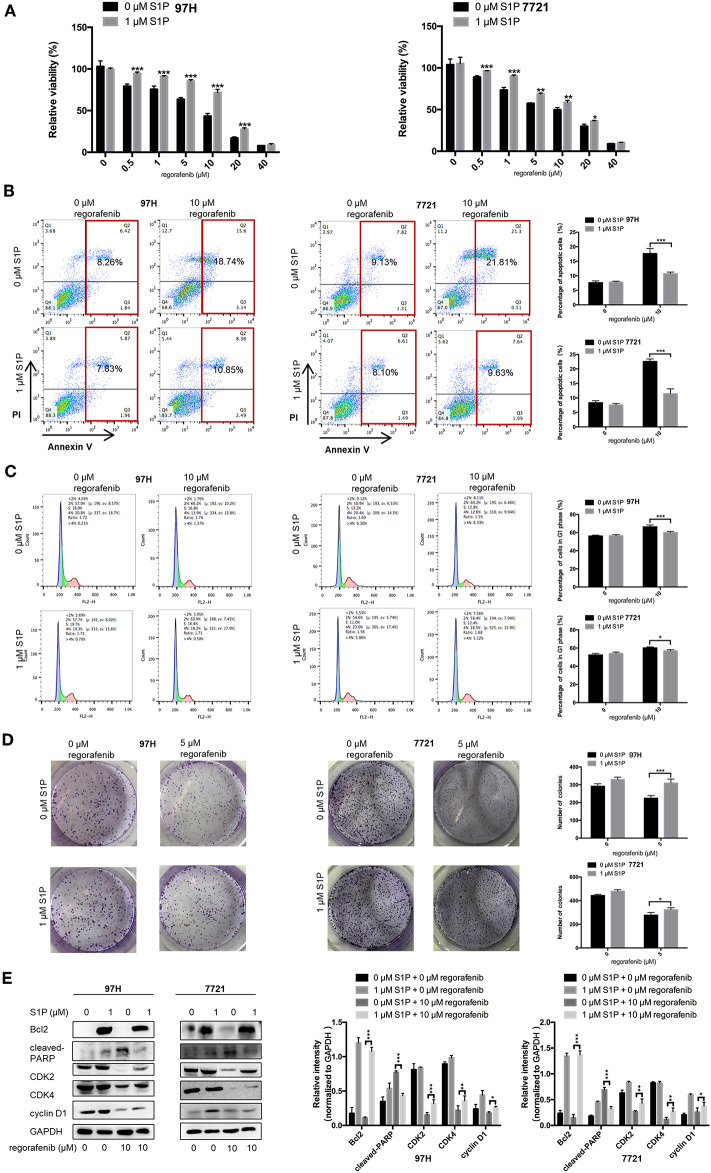
Effects of exogenous S1P on the sensitivity of HCC cells to regorafenib. **(A)** The regorafenib sensitivity of HCC cells stimulated with 1 μM S1P was examined by the CCK-8 assay. We further used the **(B)** annexin V–FITC/propidium iodide double-staining assay, **(C)** cell cycle analysis, and **(D)** colony formation assay to measure regorafenib effects on HCC cells in different groups. **(E)** The expression levels of Bcl2, cleaved PARP, cyclin D1, CDK2, and CDK4 were examined by Western blot analysis. The dose of regorafenib treatment in all assays was 10 μM for 48 h, with the exception of the colony formation assay (5 μM, 14 days for SMCC-7721 and 10 days for MHCC-97H, respectively). The result is representative for three independent experiments. The error bars represent mean ± SD from a representative experiment. *p < 0.05, **p < 0.01, ***p < 0.001.

Since the main biological function of SphK2 is to catalyze the generation of S1P, we hypothesized that the effect of SphK2 on acquired regorafenib resistance was achieved via S1P. To determine whether S1P could increase regorafenib resistance of HCC cells, we added 1 μM exogenous S1P to stimulate HCC cells. The concentration of S1P was determined based on physiological S1P content in human blood and a dose-dependent HCC cell viability analysis (Supplementary Figure 2). Sphingosine-1-phosphate stimulation decreased regorafenib sensitivity in HCC cells. The IC_50_ values of regorafenib in 97H and 7721 cells incubated with S1P were 13.13 and 10.67 μM, respectively. However, the IC_50_ values of regorafenib in 97H and 7721 cells that were not incubated with S1P were only 6.157 and 6.245 μM, respectively (Table 6). In addition, S1P stimulation decreased the influence of regorafenib on cell viability (Figure 5A), apoptosis (Figure 5B), and cell cycle progression (Figure 5C) in HCC cells. Following S1P stimulation, the proliferative potential of HCC cells was increased significantly (Figure 5D), and the changes noted in Bcl2, cleaved PARP, cyclin D1, CDK2, and CDK4 expression following regorafenib treatment of the cells were partly diminished (Figure 5E). Collectively, these data suggested that exogenous S1P stimulated the development of regorafenib resistance, indicating that SphK2 could promote regorafenib resistance in HCC by catalyzing the generation of S1P.

**Table 6:** IC_50_ values of regorafenib in S1P-stimulated HCC cells and control group cells.

**Cell line**	**Group**	**IC_**50**_ (μM)**
97H	0 μM S1P	6.157
	1 μM S1P	13.13
7721	0 μM S1P	6.245
	1 μM S1P	10.67

### Pharmacological Inhibition of SphK2 Leads to Regorafenib Sensitization in HCC Cells

**Figure 6:**
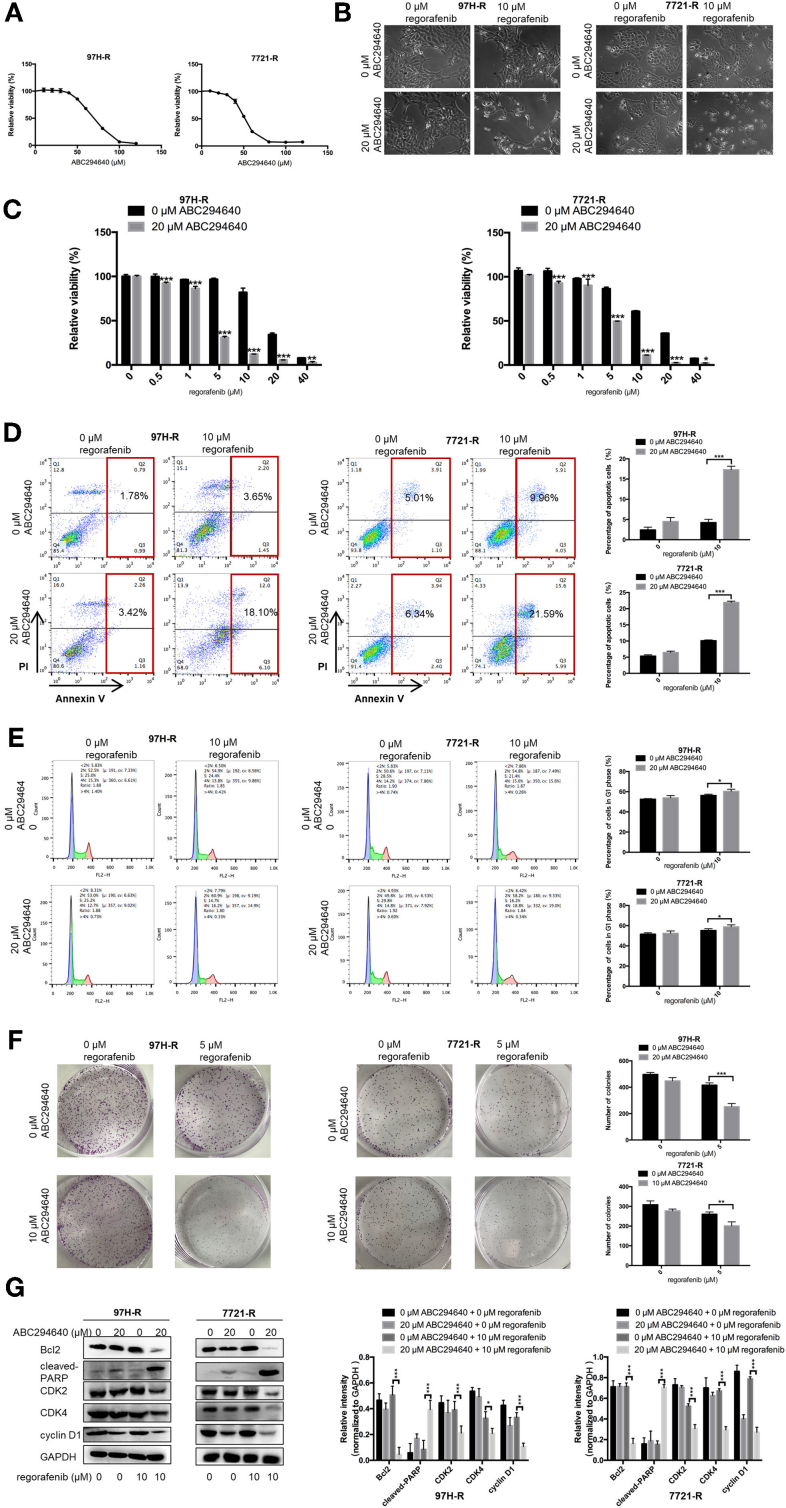
Effects of SphK2 inhibition on regorafenib resistance in HCC cells. **(A)** The effects of ABC294640 on regorafenib-resistant HCC cell viability were evaluated by the CCK-8 assay. **(B)** Regorafenib-resistant HCC cells were treated with ABC294640 and regorafenib for 48 h. Representative images of cell morphology were acquired. Subsequently, **(C)** cell viability was evaluated by the CCK-8 assay, **(D)** whereas the apoptosis rate was determined by the annexin V–FITC/propidium iodide double-staining assay. **(E)** The cell cycle distribution was measured by flow cytometry, and **(F)** cell proliferation was evaluated by the colony formation assay. **(G)** The expression levels of Bcl2, cleaved PARP, cyclin D1, CDK2, and CDK4 were examined by Western blot analysis. The doses of regorafenib and ABC294640 treatment in all assays were 10 and 20 μM, respectively, for 48 h, with the exception of the colony formation assay (5 and 10 μM, respectively, 14 days for 7721-R and 10 days for 97H-R). The result is representative for three independent experiments. The error bars represent mean ± SD from a representative experiment. *p < 0.05, **p < 0.01, ***p < 0.001.

In the present study, SphK2 was targeted by its selective inhibitor ABC294640, to evaluate the effects of SphK2 on regorafenib resistance and to explore the potential efficacy of combination treatment with regorafenib and SphK2 inhibitors. The CCK-8 assay was used to determine the effects of ABC294640 on the viability of regorafenib-resistant HCC cells. Treatment with 20 μM of ABC294640 showed little inhibition on cell viability. Therefore, this dose was selected for the SphK2 inhibitory experiments (Figure 6A). The representative images of cell morphology demonstrated that the number of cells treated with combination of ABC294640 and regorafenib was considerably decreased compared with that in other groups (Figure 6B). The CCK-8 assay results indicated that the viability of 97H-R and 7721-R cells (Figure 6C) and their regorafenib IC_50_ values (Table 7) were significantly decreased following combination treatment with ABC294640 and regorafenib for 48 h. In contrast, coadministration of selective SphK1 inhibitor PF-543 did not sensitize resistant cells to regorafenib treatment (Supplementary Figure 1D and Supplementary Table 2). Furthermore, the induction of apoptosis (Figure 6D), cell cycle arrest (Figure 6E), and the inhibition of colony formation (Figure 6F) in regorafenib-resistant cells was dramatically enhanced by concomitant exposure to ABC294640 and regorafenib, while treatment with either drug alone exhibited only marginal effects. The alterations in the expression levels of Bcl2, cleaved PARP, cyclin D1, CDK2, and CDK4 (Figure 6G) further confirmed the effects of ABC294640 on reversing regorafenib resistance. These data indicated that the application of ABC294640 could reduce regorafenib resistance of HCC cells.

**Table 7:** IC_50_ values of regorafenib in regorafenib-resistant HCC cells exposed to ABC294640 and control group cells.

**Cell line**	**Group**	**IC_**50**_ (μM)**
97H-R	0 μM ABC294640	15.99
	20 μM ABC294640	3.101
7721-R	0 μM ABC294640	12.84
	20 μM ABC294640	4.672

### The Combined Treatment of Regorafenib and ABC294640 Suppressed Tumor Growth in Xenograft Animal Model of HCC

**Figure 7:**
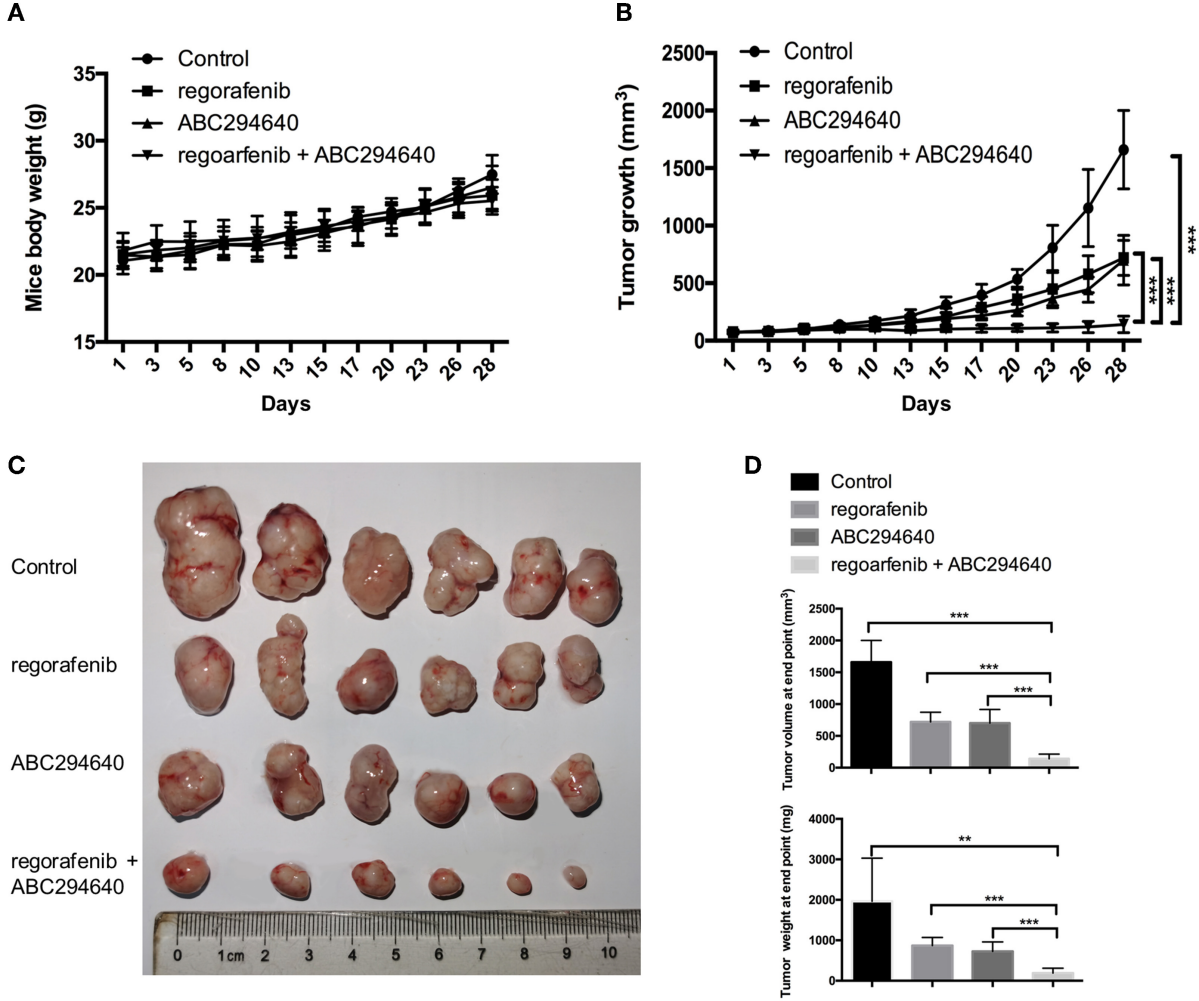
The antitumor effects of the combination treatment of ABC294640 and regorafenib in xenograft nude mice model of HCC. **(A)** Body weight of nude mice during drug treatment. **(B)** Tumor growth of subcutaneous xenograft tumors in nude mice during drug treatment. **(C)** Image of tumors harvested from nude mice treated with vehicle, regorafenib, ABC294640, and the combination with regorafenib and ABC294640. **(D)** Average volume and weight of subcutaneous xenograft tumors harvested from nude mice at the end point of the study. The error bars represent mean ± SD. **p < 0.01, ***p < 0.001.

The present study further evaluated the potential therapeutic efficacy of combination treatment with ABC294640 and regorafenib in a nude mice xenograft model established by regorafenib-resistant MHCC97H cells. There was no significant difference in body weight between mice treated with drugs and mice treated with vehicle, and no mice died during the treatment, indicating little systemic toxicity of these drugs (Figure 7A). As shown in Figures 7B,C, compared with vehicle control, treatment of either regorafenib or ABC294640 showed mild tumor inhibitory effects, whereas combination treatment with both drugs dramatically suppressed the growth of tumor. The measurement of the volume and weight (Figure 7D) of tumors also confirmed that coadministration of AB294640 with regorafenib sensitized the resistant cells to treatment.

### SphK2/S1P Regulates Regorafenib Resistance of HCC Cells by Inducing the Activation of Nuclear Factor κB and Signal Transducer and Activator of Transcription 3

**Figure 8:**
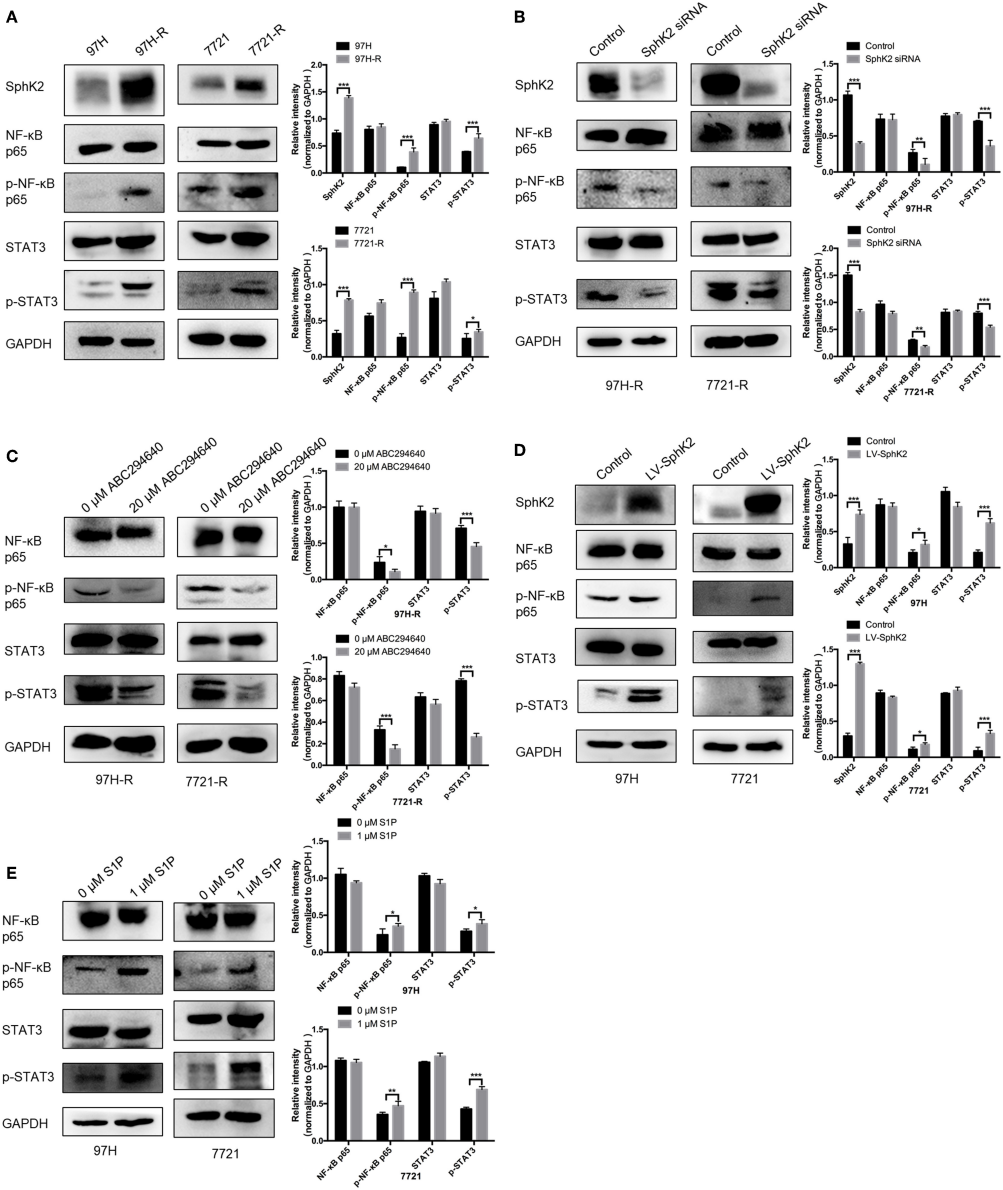
Phosphorylation of NF-κB p65 and STAT3 in HCC cells. **(A)** The phosphorylation of NF-κB p65 and STAT3 in parental and regorafenib-resistant HCC cells was examined by Western blot analysis. **(B)** The phosphorylation of NF-κB p65 and STAT3 in regorafenib-resistant HCC cells transfected with SphKK2 siRNA. **(C)** The phosphorylation of NF-κB p65 and STAT3 in regorafenib-resistant HCC cells treated with ABC294640. **(D)** The phosphorylation of NF-κB p65 and STAT3 in SphK2-overexpressing parental HCC cells. **(E)** The phosphorylation of NF-κB p65 and STAT3 in HCC cells exposed to S1P. The density of each band was measured and normalized to respective GAPDH. The result is representative for three independent experiments. The error bars represent mean ± SD from a representative experiment. *p < 0.05, **p < 0.01, ***p < 0.001.

Currently, the molecular mechanisms of SphK2-mediated regorafenib resistance in HCC remain unknown. Because nuclear factor κB (NF-κB) and signal transducer and activator of transcription 3 (STAT3) are targets of regorafenib and ABC294640, we hypothesized that SphK2/S1P could regulate regorafenib resistance in HCC cells through NF-κB and STAT3 activation. Therefore, we determined the phosphorylation levels of NF-κB p65 and STAT3 in HCC cells following different treatments. Western blot analysis indicated that the phosphorylation levels of NF-κB p65 and STAT3 were increased in regorafenib-resistant HCC cells (Figure 8A) and SphK2-overexpressing HCC cells (Figure 8D), whereas they were decreased in regorafenib-resistant HCC cells following SphK2 knockdown or inhibition (Figures 8B,C). In addition, exogenous S1P treatment promoted the phosphorylation of NF-κB p65 and STAT3 (Figure 8E). Based on these experimental data, the present study confirmed that NF-κB and STAT3 activation was involved in SphK2/S1P-mediated regorafenib resistance in HCC cells.

## Discussion

Chemoresistance is a complex process that develops in the majority of cancer types and causes poor therapeutic responses along with treatment failure (27). Regorafenib is a crucial drug for treating metastatic colorectal cancer (CRC), advanced gastrointestinal stromal tumors, and HCC (28). To date, only a limited number of studies have been carried out on regorafenib resistance, and most in CRC (5). This is the first study that demonstrated SphK2/S1P was the key regulator in mediating regorafenib resistance of HCC by the activation of NF-κB and STAT3. Moreover, ABC294640, a selective inhibitor of SphK2, exhibited high potential to increase the sensitivity of regorafenib-resistant HCC cells to regorafenib.

Dysregulation of sphingolipid metabolism and signaling has recently been shown to be associated with chemoresistance. Lower levels of ceramide and higher levels of S1P were simultaneously observed in gemcitabine-resistant pancreatic cancer cells than in gemcitabine-sensitive pancreatic cancer cells, whereas increasing ceramide concentrations or decreasing S1P concentrations sensitized pancreatic cancer cells to gemcitabine-induced cell death (29). In addition, the ceramide: S1P ratio was also decreased in docetaxel-resistant cells (30) and imatinib-resistant cells (31). In the present study, SphK2, the rate-limiting enzyme in sphingolipid metabolism, was found to play a vital role in regorafenib resistance in HCC. In well-established regorafenib-resistant HCC cells, the expression levels of SphK2 were substantially higher than those in parental cells. Significantly increased SphK2 expression levels were also observed in chemoresistant breast cancer cells (32). In addition, a negative correlation between SphK2 protein levels and the sensitivity to regorafenib was noted in the five HCC cell lines. These results were similar to those reported from the study by Yang et al. (20) demonstrating that SphK2 expression correlated negatively with tumor necrosis factor-related apoptosis-inducing ligand (TRAIL) sensitivity in three NSCLC cell lines.

To further investigate the role of SphK2 in promoting regorafenib resistance in HCC, we inhibited the expression of SphK2 in regorafenib-resistant cells and increased the SphK2 expression in normal HCC cells. Knockdown of SphK2 restored regorafenib sensitivity of regorafenib-resistant HCC cells. A previous study demonstrated that knockdown of SphK2 could induce the apoptosis of gefitinib-resistant NSCLC cells, which is consistent with the results reported in present study (13). Our results also showed that overexpression of SphK2 increased regorafenib resistance of normal HCC cells. Consistently, Shi et al. (16) reported that the increased levels of SphK2 expression led to ATRA therapy resistance in human colonic adenocarcinoma HCT-116 cells. Taking these data together, we demonstrated the important role of SphK2 in mediating regorafenib resistance in HCC, which could be a potential target to overcome regorafenib resistance.

In the present study, we further explored the role of SphK1 in regorafenib resistance of HCC cells. There was no obvious difference in the expression levels of SphK1 between parental cells and regorafenib-resistant cells. In addition, inhibition of SphK1 did not restore the sensitivity of HCC cells to regorafenib. These results indicated that SphK1 may not be involved in regorafenib resistance, and SphK2 was the specific SphK mediating regorafenib resistance.

Because S1P is the main product of SphK2, the present study revealed that S1P mediates regorafenib resistance in HCC cells. Accumulating evidence demonstrated that in addition to its role as a sphingosine metabolite, S1P is a critical secondary messenger that mediates chemoresistance (30). A high level of S1P was detected in camptothecin-resistant PC-3 prostate cancer cells, and inhibition of the S1P receptor signaling significantly decreased cell growth (33). Sphingomab, a neutralizing antibody against S1P, also showed inhibitory effect on sunitinib-resistant renal carcinoma cell growth (34). The present data indicated that the supplementation of S1P reduced the sensitivity of HCC cells to regorafenib. However, the IC_50_ values of regorafenib in HCC cells stimulated with S1P were not as high as those in HCC cells with acquired regorafenib resistance. Possibly, the intracellular S1P levels in HCC cells stimulated with 1 μM S1P were different from those in regorafenib-resistant cells. In previous studies (35), the doses of S1P used to stimulate HCC cells were 1, 3, and 10 μM, which were all higher than that used in our study. Alternatively, exogenous addition of S1P may not influence endogenous ceramide production, which has inhibitory effects on chemoresistance of HCC cells. In contrast to the effects of S1P on chemoresistance, exogenous addition of ceramide (1 μM) to pancreatic cancer cells increased their sensitivity to gemcitabine (30). In addition, it has been shown that 20 μM S1P treatment exhibited no effects on ceramide production in leukemia HL-60 cells (36). Therefore, we hypothesized that the ratio of ceramide to S1P in HCC cells treated with 1 μM S1P was not as low as that in HCC cells with acquired regorafenib resistance. Subsequently, we will further explore the changes and the function of complex sphingolipid metabolism in regorafenib resistance in HCC.

As we demonstrated the promoting effect of SphK2/S1P on the regorafenib resistance of HCC cells, we aimed to reverse regorafenib resistance by targeting SphK2. ABC294640 is a novel selective inhibitor of SphK2 that has been found to exert broad anticancer activity. The application of ABC294640 enhanced the effects of specific antitumor drugs, such as TRAIL-induced apoptosis in NSCLC cells (20) and paclitaxel-induced apoptosis in Caov-3 ovarian cancer cells (37). The present study is the first to explore the biological effects of ABC294640 on regorafenib-resistant HCC cells. We investigated the efficacy of the combination treatment with ABC294640 and regorafenib toward regorafenib-resistant HCC. The combination of ABC294640 with regorafenib increased the induction of apoptosis and decreased the proliferation of regorafenib-resistant HCC cells. The dose of ABC294640 used in our study was 20 μM in most assays and 10 μM in the colony formation assay, which was considerably lower than that used in previous studies (37). By using 20 or 10 μM ABC294640 alone, only a mild effect was noted on proliferation and apoptosis of regorafenib-resistant cells. However, the combination of regorafenib with ABC294640 remarkably inhibited cell proliferation and promoted apoptosis in regorafenib-resistant HCC cell lines *in vitro*. In addition, the combined treatment of regorafenib and ABC294640 suppressed tumor growth of HCC resistant cells in a xenograft tumor model. The combination significantly reduced the volume and weight of developed tumors compared to individual treatments and the vehicle control animals. These findings suggest the potential of the SphK2 inhibitor ABC294640 to reverse regorafenib resistance and provide a high clinical value for the treatment of regorafenib-resistant HCC patients.

Although we demonstrated that SphK2/S1P plays important roles in mediating regorafenib resistance in HCC, the molecular mechanism of its action remains unclear. The present study indicated that NF-κB and STAT3 were involved in regorafenib resistance and that they were the downstream effectors of SphK2/S1P signaling. Western blot analysis demonstrated that the phosphorylation levels of NF-κB p65 and STAT3 were higher in regorafenib-resistant cells compared to parental cells. In addition, SphK2 overexpression and S1P addition increased the phosphorylation levels of NF-κB p65 and STAT3, which were decreased following inhibition of SphK2. The involvement of NF-κB in chemoresistance has been reported in doxorubicin-resistant breast cancer cells (38). In addition, STAT3 is significantly activated in sorafenib-resistant cells (39) and multidrug-resistant myeloma cells (40). Our findings further support previous evidence indicating that NF-κB and STAT3 are important mediators of chemoresistance. In addition, the results indicating that NF-κB and STAT3 are the downstream effectors of SphK2/S1P signaling have been observed in earlier studies. For example, S1P promoted the activation of STAT3 in cardiomyocytes (41), and ABC294640 blocked NF-κB activity in multidrug-resistant breast cancer cells (32).

## Conclusions

Collectively, our data indicated that SphK2/S1P mediated regorafenib resistance of HCC cells. The activation of NF-κB and STAT3 played an important regulatory role in regorafenib resistance. The two key proteins serve as downstream effectors of SphK2/S1P, elucidating a novel mechanism, which links SphK2/S1P to NF-κB and STAT3 in regorafenib-resistant HCC cells. Most notably, the combination treatment of regorafenib with ABC294640 inhibited the proliferation and promoted the apoptosis of regorafenib-resistant HCC cells, providing new insights to overcome acquired resistance to regorafenib treatment and enhancing therapeutic outcomes for patients with advanced HCC.

## Materials and Methods

### Cell Culture, Antibodies, Chemicals, and Reagents

The human HCC cell lines BEL-7402, HuH-7, PLC/PRF/5, and SMMC-7721 were purchased from the Cell Bank of the Chinese Academy of Sciences (Shanghai, China). The HCC cell line MHCC97H was kindly provided by Prof. Jia Fan from Zhongshan Hospital of Fudan University. The cells were maintained in Dulbecco modified Eagle medium (DMEM) supplemented with 10% fetal bovine serum (FBS), 100 U/mL penicillin, and 100 μg/mL streptomycin sulfate in a humidified incubator at 37°C in an atmosphere containing 5% CO_2_ in air. The suppliers and catalog numbers of all antibodies, chemicals, and reagents used in this study are listed in Supplementary Table 3.

### Establishment of Acquired Resistance to Regorafenib

Resistant cell lines (7721-R and 97H-R) were established by treating cells with stepwise increasing concentrations of regorafenib. The protocol was modified from the establishment of sorafenib-resistant cell lines described in a previous study (42). Briefly, 1 × 10^6^ cells were cultured in 6-mm plates and were incubated with regorafenib at a concentration just below their respective IC_50_ (5 μM for both SMCC-7721 and MHCC97H). The concentration of regorafenib was slowly increased by 0.5 μM per week. Dead cells were washed, and viable cells were cultured in fresh medium containing stepwise increasing concentrations of regorafenib. In parallel, control wild-type cells were treated with the corresponding vehicle. After 6 months, the IC_50_ to regorafenib was determined to confirm the establishment of regorafenib-resistant HCC cells. Regorafenib-resistant HCC cells were continuously maintained by culturing them in the presence of 4 μM regorafenib.

### siRNA Transfection

SphK2 and SphK1 expression was downregulated by transfection with sequence-specific siRNAs. siRNA against human SphK2 (targeted sequence: GGGUAGUGCCUGAUCAAUGTT, 5′ to 3′), human SphK1 (targeted sequence: GGGCAAGGCCUUGCAGCUCTT, 5′ to 3′), and scrambled control siRNA (GenePharma, Shanghai, China) were used. The siRNAs were diluted in 150 μL of OptiPro™ SFM. Lipofectamine™ 2000 (Thermo Fisher Scientific, Waltham, MA, USA) was mixed gently before use, and the appropriate amount was subsequently diluted in 150 μL of OptiPro™ SFM. The solution was incubated for 5 min at room temperature. Following 5 min of incubation, the diluted DNA was combined with diluted Lipofectamine™ 2000 (total volume = 300 μL). The solution was mixed gently for 20 min at room temperature. The complexes (300 μL) were added to a six-well dish containing cells and medium. The cells were incubated at 37°C in a CO_2_ incubator for 18 to 48 h prior to further assays.

### Lentiviral Transfection

Lentiviral transfection was used to obtain HCC cells with stable ectopic SphK2 overexpression. Lentivirus expressing SphK2 and corresponding negative control virus, both with puromycin resistant gene, were purchased from Shanghai GeneChem Company Ltd. (Shanghai, China). Hepatocellular carcinoma cells were plated in 6-well plates at a density of 2 × 10^5^ cells per well and were subsequently transfected with lentivirus at a multiplicity of infection of 10. Following 48 h of incubation, the antibiotic-resistant transfected cells were selected and enriched by applying culture medium containing puromycin.

### CCK-8 Assay

For CCK-8 assay, the powder of regorafenib, ABC294640, and S1P were dissolved in dimethyl sulfoxide (DMSO) to make stock solutions containing 50, 100, and 10 mM indicated chemicals, respectively. The final concentration of DMSO in the treatment medium was <0.1%. Hepatocellular carcinoma cells in DMEM containing 10% FBS were seeded into 96-well plates at a concentration of 1 × 10^4^ cells per well and incubated for 24 h. The culture medium was replaced with fresh medium containing vehicle or testing reagents at indicated concentrations. After treating cells with different reagents or vehicle for 48 h, CCK-8 solution (10 μL/well) was added to the 96-well plates and incubated for 1 h to detect the viability of HCC cells. The absorbance values at 450 nm were measured in a microplate reader (Bio-Rad, Hercules, CA, USA), and cell viability was determined. Relative viability was normalized to the vehicle-treated control cells after background subtraction and was expressed as OD_test_/OD_control_ × 100%. The IC_50_ value was defined as the drug concentration that inhibits 50% cell viability compared with vehicle-treated controls and calculated by GraphPad Prism 6.0 software (GraphPad Software, La Jolla, CA, USA). Each treatment was performed in triplicate wells, and three independent repeats of experiments were performed.

### Colony Assay

Hepatocellular carcinoma cells were plated in 6-well plates at a density of 1 × 10^3^ cells per well in DMEM containing 10% FBS and allowed to adhere overnight. The culture media was replaced with fresh media containing vehicle or testing reagents every 3 days. The concentration of regorafenib, ABC294640 and S1P for colony assay was 5, 10, and 1 μM, respectively. After incubation (14 days for SMCC-7721 and 7721-R, 10 days for MHCC-97H and 97H-R), the cells were washed and fixed with 4% paraformaldehyde (20 min). The plates were incubated with 0.4% crystal violet solution (30 min) and washed with phosphate-buffered saline (PBS) and dried. The total number of colonies (≥30 cells) (32) in each well was counted manually. Three independent repeats of experiments were performed.

### Cell Cycle Assay

The cell cycle distribution of different cells was determined by flow cytometry. The cells (approximately 1 × 10^6^ cells per well) were harvested following different treatments and fixed overnight in 70% ethanol at 4°C. Following fixation, the cells were centrifuged at 1,000 × g for 5 min to remove the ethanol, washed, and stained with propidium iodide (PI) (10 μg/mL) and RNase A (100 μg/mL) at room temperature for 30 min. Propidium iodide detection was achieved with a BD FACSCalibur flow cytometer (Becton–Dickinson, San Jose, CA, USA). The distribution of the cells in the different phases of the cell cycle was analyzed and calculated using the FlowJo software (Tree star, San Carlos, CA, USA). The blue, green, and red parts in the figure of cell cycle distribution represent cells in G1 phase, S phase, and G2/M phase, respectively. Three independent repeats of experiments were performed.

### Annexin V–Fluorescein Isothiocyanate/PI Double-Staining Assay

Cells (approximately 1 × 10^6^ cells per well) were collected and centrifuged at 1,000 × g for 5 min at room temperature, resuspended in ice-cold PBS, centrifuged at 1,000 × g for 5 min, and washed. The cells were resuspended by adding 500 μL of 1 × binding buffer. Subsequently, 5 μL annexin V–fluorescein isothiocyanate (FITC) staining solution and 5 μL PI staining solution were added to the suspension, mixed well, and incubated for 30 min at room temperature. Fluorescence intensity was measured using a BD FACSCalibur cytometer (Becton–Dickinson), and the apoptotic rates of cells were analyzed using the FlowJo software (Tree Star). The cells in Q1 (left upper quadrant), Q2 (right upper quadrant), Q3 (right lower quadrant), and Q4 (left lower quadrant) represent dead cells, late apoptotic cells, early apoptotic cells, and living cells, respectively. Three independent repeats of experiments were performed.

### Protein Isolation and Western Blot Analysis

The cells were lysed with 150 μL lysis buffer (Beyotime, Shanghai, China) containing 1% protease inhibitors (Thermo Fisher Scientific) on ice for 5 min following washing twice with ice-cold PBS. The cells were harvested and centrifuged at 12,000 × g for 5 min at 4°C. The protein concentrations were determined using a BCA Kit (Beyotime). Equal amounts of protein (20 μg/lane) dissolved in 20 μL loading buffer (Beyotime) were separated by sodium dodecyl sulfate–polyacrylamide gel electrophoresis (Beyotime), transferred to polyvinylidene difluoride (Roche Applied Science, Mannheim, Germany) membranes, and blocked with 5% non-fat dry milk for 1 h at room temperature. Immunoblotting was carried out by incubation overnight at 4°C with the indicated primary antibodies. Catalog numbers and suppliers of antibodies used are listed in Supplementary Table 3. The dilution of primary antibodies against SphK1 and SphK2 was 1:500. Other primary antibodies were diluted at 1:1,000. After the incubation with primary antibodies, membranes were washed and incubated with HRP-linked secondary antibodies (1:5,000 dilution) at room temperature for 1 h. The signals were developed with an enhanced chemiluminescence reagent (Biosharp, Beijing, China) under a chemiluminescence camera (Tanon, Beijing, China). The density of each band was measured using ImageJ software (National Institutes of Health, Maryland, MD, USA) and was normalized to internal loading control (GAPDH) from the same sample. Three independent repeats of experiments were performed.

### Tumor Xenograft Model

Six-week-old male BALB/c nude mice, weighing ~20 g, were purchased from the Model Animal Research Center of Nanjing University. Mice were housed in sterile cages in laminar airflow hoods in a specific pathogen-free environment at 22 to 25°C, relative humidity 40°C to 60% with a 12:12-h day–night light cycle. The mice had free access to autoclaved water and commercial mice food (Xietong Biological, Nanjing, China). The protocol of this study was approved by the Institutional Ethics Committee of the Affiliated Drum Tower Hospital of Nanjing University Medical School. Regorafenib-resistant MHCC-97H (97H-R) cells (1 × 10^7^ cells in 100 μL PBS) were injected subcutaneously to nude mice. When the long diameter of tumors reached 5 mm, mice were randomly assigned to four groups (n = 6 per group) and were orally treated with vehicle, regorafenib, ABC294640, or both regorafenib and ABC294640, respectively. Regorafenib and ABC294640 were suspended in an oral vehicle containing 2% DMSO + 30% PEG300 (Selleck, Mattapoisett, MA, USA) + 5% Tween 80 (Selleck) + ddH_2_O. Mimicking the clinically recommended administration schedule of regorafenib in human, 20 mg/kg regorafenib was given orally once daily for the first 21 days. ABC294640 was given 40 mg/kg orally, three times a week for 4 weeks according to a previously published study (43). Tumors were measured with a digital caliper three times a week, and the volume was calculated by the formula: length × width^2^ × 0.5. The body weight of animals was also measured three times a week. All mice were sacrificed by cervical dislocation under general anesthesia with isoflurane (RWD Life Science, Shenzhen, China) after 4 weeks of treatment, and the tumors were harvested.

### Statistical Analysis

The data were analyzed using SPSS 19.0 statistical software (IBM, Chicago, IL, USA) and expressed as the mean ± SD of a representative independent experiment. The comparisons between two groups were performed with Student *t*-test, whereas the comparisons among multiple groups were performed with one-way analysis of variance followed by the Student–Newman–Keuls *post-hoc* test. *P* < 0.05 was considered to indicate a statistically significant difference.

## Data Availability Statement

All datasets generated for this study are included in the article/Supplementary Material.

## Ethics Statement

The animal study was reviewed and approved by Institutional Ethics Committee of the Affiliated Drum Tower Hospital of Nanjing University Medical School.

## Author Contributions

CJ, JW, ZW, and WS conceived and designed the experiments. WS, SZ, DM, DY, GZ, and YC performed the experiments. WS, SZ, DM, DY, GZ, and YC analyzed the data. WS and SZ wrote the original manuscript. JW and ZW reviewed and edited the manuscript. CJ, JW, and ZW acquired the funding. All authors contributed to the article and approved the submitted version.

## Conflict of Interest

The authors declare that the research was conducted in the absence of any commercial or financial relationships that could be construed as a potential conflict of interest.
