# Supplementary material for: Targeting SphK2 Reverses Acquired Resistance of Regorafenib in Hepatocellular Carcinoma
Source: Front Oncol. 2020 Jun 24;10:694. doi: 10.3389/fonc.2020.00694 (PMC7327090; doi:10.3389/fonc.2020.00694)
Supplement: Supplementary file 1 [file Data_Sheet_1.pdf]

Targeting SphK2 reverses acquired resistance of regorafenib in hepatocellular carcinoma

Weiwei Shi[1, 2, #], Shan Zhang[1, 2, #], Ding Ma[1, 2], Dongliang Yan[3], Guang Zhang[1, 2, 3], Yin Cao[1, 2, 3], Zhongxia Wang[1, 2, 3, *], Junhua Wu[2, *], Chunping Jiang[1, 2, 3, *].

[1]Department of Hepatobiliary Surgery, the Affiliated Drum Tower Hospital of Nanjing University Medical School, Nanjing, Jiangsu 210008, China
[2]Jiangsu Key Laboratory of Molecular Medicine, Medical School, Nanjing University, Nanjing, Jiangsu 210093, China
[3]Department of Hepatobiliary Surgery, Drum Tower Clinical College of Nanjing Medical University, Nanjing, Jiangsu 210008, China

[#] These authors contributed equally to this work.

*** Correspondence:**

Chunping Jiang. chunpingjiang@163.com.

Junhua Wu. wujunhua@nju.edu.cn.

Zhongxia Wang. freud_t@126.com.

Supplementary Figure 1

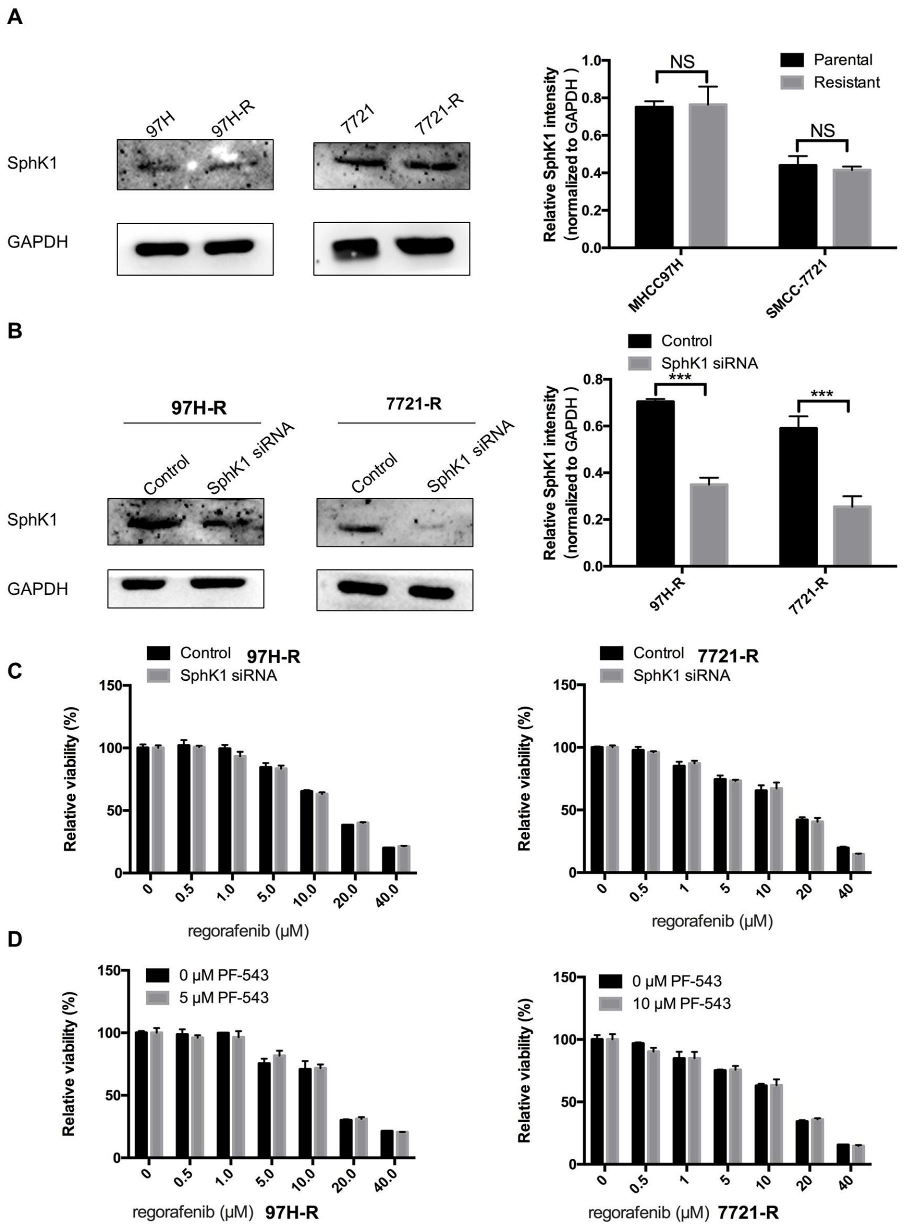

Supplementary Figure 1. The role of SphK1 in regorafenib-resistant HCC cells. (A) SphK1 protein expression levels in parental and regorafenib-resistant HCC cells were determined by western blot analysis. **(B)** The protein expression levels of SphK1 in regorafenib-resistant HCC cells transfected with SphK1 siRNA or control siRNA were determined by western blot analysis. **(C)** Regorafenib-resistant HCC cells were transfected with SphK1 siRNA or control siRNA and treated with regorafenib. Cell viability was determined by the CCK-8 assay. **(D)** Cell viability of regorafenib-resistant HCC cells treated with regorafenib alone or together with PF-543 was determined by the CCK-8 assay. The results were obtained from three independent experiments. The error bars represent mean ± SD from a representative experiment. *** $p<0.001$, NS, not significant.

Supplementary Figure 2

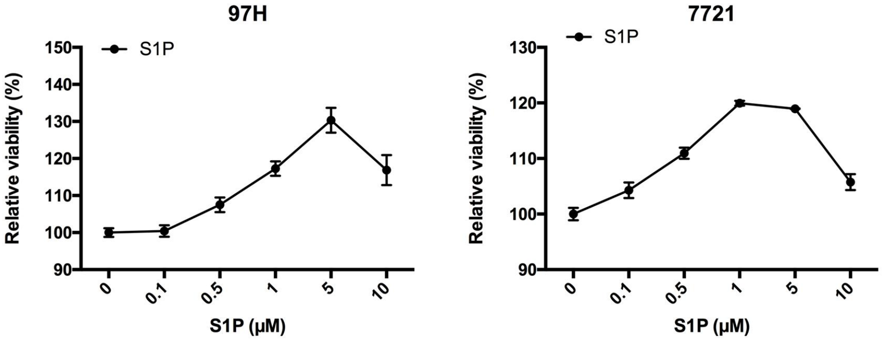

Supplementary Figure 2. The effects of S1P on growth of HCC cells. The effect of S1P on HCC cell viability was evaluated by the CCK-8 assay.

Supplementary Table 1. IC_{50} values of regorafenib in regorafenib-resistant HCC cells transfected with control or SphK1 siRNA.

Cell line	Group	IC_{50} (μM)
97H-R	Control	15.11
	SphK1 siRNA	15.08
7721-R	Control	14.31
	SphK1 siRNA	13.76

Supplementary Table 2. IC_{50} values of regorafenib in regorafenib-resistant HCC cells exposed to vehicle or SphK1 inhibitor PF-543.

Cell line	Group	IC_{50} (μM)
97H-R	0 μM PF-543	14.01
	5 μM PF-543	14.70
7721-R	0 μM PF-543	12.61
	10 μM PF-543	12.50

Supplementary Table 3. The suppliers and catalog numbers of all chemical compounds used in the study.

REAGENTS	SUPPLIER	CATALOG NUMBER
Antibodies		
Rabbit monoclonal anti-Bcl2	Cell Signaling Technology (MA, USA)	Cat# 2870P
Rabbit monoclonal anti-cleaved-PARP	Cell Signaling Technology (MA, USA)	Cat# 5625P
Rabbit monoclonal anti-CDK4	Cell Signaling Technology (MA, USA)	Cat# 2906P
Rabbit monoclonal anti-CDK6	Cell Signaling Technology (MA, USA)	Cat# 3136P
Rabbit monoclonal anti-cyclin D1	Cell Signaling Technology (MA, USA)	Cat# 2978S
Rabbit monoclonal anti-STAT3	Cell Signaling Technology (MA, USA)	Cat# 4904S
Mouse monoclonal anti-p-STAT3	Cell Signaling Technology (MA, USA)	Cat# 4113S
Rabbit monoclonal anti-NF-κB p65	Cell Signaling Technology (MA, USA)	Cat# 8242S
Rabbit monoclonal anti-p-NF-κB p65	Cell Signaling Technology (MA, USA)	Cat# 3033S
Rabbit monoclonal anti-SphK1	ProteinTech Group (Wuhan, China)	Cat# 10670-1-AP
Rabbit monoclonal anti-SphK2	ProteinTech Group (Wuhan, China)	Cat# 17096-1-AP
Anti-rabbit IgG, HRP-linked antibody	Cell Signaling Technology (MA, USA)	Cat# 7074
Anti-mouse IgG, HRP-linked antibody	Cell Signaling Technology (MA, USA)	Cat# 7076
Chemicals		
ABC294640	Selleck Chemicals (MA, USA)	Cat# 915385-81-8
Crystal violet	Sigma-Aldrich (MO, USA)	Cat# 548-62-9
Dimethyl sulfoxide（DMSO）	Sigma-Aldrich (MO, USA)	Cat# 67-68-5
Dulbecco's modified Eagle's medium (DMEM)	WISENT (Nanjing, China)	Cat# 319-005-CL
Fetal bovine serum	WISENT (Nanjing, China)	Cat# 087-150
Isoflurane	RWD Life Science (Shenzhen, China)	Cat# 217170701
Lipofectamine™ 2000	Life Technologies (CA, USA)	Cat# 11668019
NP-40 lysis buffer	Beyotime (Shanghai, China)	Cat# P0013F
OptiPro™ SFM	Life Technologies (CA, USA)	Cat# 12309019
Paraformaldehyde	Sigma-Aldrich (MO, USA)	Cat# p6148
Penicillin and streptomycin	WISENT (Nanjing, China)	Cat# 450-201-EI
PMSF Protease Inhibitor	Thermo Fisher Scientific (MA, USA)	Cat# 36978
Polyvinylidene difluoride (PVDF) membranes	Roche Applied Science (Mannheim, Germany)	Cat# 03010040001
Primary Antibody Dilution Buffer	Beyotime (Shanghai, China)	Cat# P0023A
Regorafenib	Selleck Chemicals (MA, USA)	Cat# S1178
Sodium dodecyl sulfate polyacrylamide gel electrophoresis (SDS-PAGE)	Beyotime (Shanghai, China)	Cat# P0690
Sphingosine-1-Phosphate (S1P)	Cayman Chemical (MI, USA)	Cat# 62570
High-Sig ECL Western Blotting Substrate	Biosharp (Beijing, China)	Cat# BL520A
Critical Assays		
Annexin V-FITC/PI double staining assay kit	Vazyme (Nanjing, China)	Cat# A211-02
BCA protein assay Kit	Beyotime (Shanghai, China)	Cat# P0010
Cell Counting Kit-8 assay kit	Selleck Chemicals (MA, USA)	Cat# B34304
Cell cycle assay kit	MultiSciences (Hangzhou, China)	Cat# 70-CCS012
Cell Lines		
BEL-7402 human HCC cells	Cell Bank of the Chinese Academy of	TCHu10

	Sciences (Shanghai, China)	
HuH-7 human HCC cells	Cell Bank of the Chinese Academy of Sciences (Shanghai, China)	TCHu182
MHCC97H human HCC cells	Professor Jia Fan from Zhongshan Hospital of Fudan University	N/A
PLC/PRF/5 human HCC cells	Cell Bank of the Chinese Academy of Sciences (Shanghai, China)	TCHu119
SMMC-7721 human HCC cells	Cell Bank of the Chinese Academy of Sciences (Shanghai, China)	TCHu52
Experimental Models		
Mouse: BALB/c nu/nu	Model Animal Research Center of Nanjing University (Nanjing, China)	N/A
Commercial mice food	Xietong Biological (Nanjing, China)	Cat# 1010010
